# Trazodone plus pregabalin combination in the treatment of fibromyalgia: a two-phase, 24-week, open-label uncontrolled study

**DOI:** 10.1186/1471-2474-12-95

**Published:** 2011-05-16

**Authors:** Elena P Calandre, Piedad Morillas-Arques, Rocío Molina-Barea, Carmen M Rodriguez-Lopez, Fernando Rico-Villademoros

**Affiliations:** 1 Instituto de Neurociencias y Centro de Investigaciones Biomédicas, Universidad de Granada, Granada, Spain

## Abstract

**Background:**

Although trazodone is frequently used by fibromyalgia patients, its efficacy on this disease has not been adequately studied. If effective, pregabalin, whose beneficial effects on pain and sleep quality in fibromyalgia have been demonstrated, could complement the antidepressant and anxiolytic effects of trazodone. The aim of the present study was to assess the effectiveness of trazodone alone and in combination with pregabalin in the treatment of fibromyalgia.

**Methods:**

This was an open-label uncontrolled study. Trazodone, flexibly dosed (50-300 mg/day), was administered to 66 fibromyalgia patients during 12 weeks; 41 patients who completed the treatment accepted to receive pregabalin, also flexibly dosed (75-450 mg/day), added to trazodone treatment for an additional 12-week period. Outcome measures included the Fibromyalgia Impact Questionnaire (FIQ), the Pittsburgh Sleep Quality Index (PSQI), the Beck Depression Inventory (BDI), the Hospital Anxiety and Depression Scale (HADS), the Brief Pain Inventory (BPI), the Short-Form Health Survey (SF-36), and the Patients' Global Improvement scale (PGI). Emergent adverse reactions were recorded. Data were analyzed with repeated measures one-way ANOVA and paired Student's t test.

**Results:**

Treatment with trazodone significantly improved global fibromyalgia severity, sleep quality, and depression, as well as pain interference with daily activities although without showing a direct effect on bodily pain. After pregabalin combination additional and significant improvements were seen on fibromyalgia severity, depression and pain interference with daily activities, and a decrease in bodily pain was also apparent. During the second phase of the study, only two patients dropped out due to side effects.

**Conclusions:**

Trazodone significantly improved fibromyalgia severity and associated symptomatology. Its combination with pregabalin potentiated this improvement and the tolerability of the drugs in association was good.

**Trial Registration:**

ClinicalTrials.gov: NCT00791739

## Background

Disturbed sleep is a prominent feature of fibromyalgia symptomatology and has been proposed as one of the core symptoms that should be systematically assessed in clinical trials for the treatment of fibromyalgia [1]. Trazodone is an old second-generation antidepressant with strong sedative activity, widely used as a hypnotic drug in subtherapeutic antidepressant doses of 100 mg or less, although the evidence of the efficacy of this drug in treating insomnia in nondepressed patients is very limited [2]. Despite this relative paucity of data, low-dose trazodone is frequently used in fibromyalgia management to improve sleep quality [3]. In a recent Internet survey of people with fibromyalgia, 33% of the patients reported having used trazodone; within this group, the 36% of respondents reported continuing to use the drug, with 51% finding it useful [4]. In fact, as symptoms of depression and anxiety are frequently found in fibromyalgia patients, the acknowledged antidepressant and anxiolytic properties of trazodone [5] could be useful in treating other symptoms of fibromyalgia in addition to insomnia.

However, the efficacy of trazodone for fibromyalgia, either as a hypnotic or as an antidepressant, has not been adequately investigated. Only one study, which was published in abstract form, evaluated the polysomnographic and clinical effects of trazodone in women with fibromyalgia [6]. The authors stated that two-month treatment with trazodone increased slow-wave sleep and reduced alpha activity but did not improve pain or psychological distress. However, their study included only 13 patients and they did no mention the daily dosage of trazodone that was used.

Although monotherapy would be the optimal treatment approach in fibromyalgia, the multidimensional nature of the disease and its high comorbidity with other disorders often leads to the use of polypharmacy. Patients with a wide range of symptoms frequently require the simultaneous use of more than one drug, and the need to prescribe two or more drugs for fibromyalgia management has been acknowledged [7,8]. In fact, several observational, prospective and longitudinal studies have found that the use of prescription drugs is high among fibromyalgia patients [9], and that polytherapy is usual, with the mean number of drugs ranging from 2.7 to 3.3 per patient [10-12]. Therefore, combination therapy, using drugs targeting different symptoms of the disease, seems worthy of being explored.

Pregabalin was the first drug to be approved by the FDA for the treatment of fibromyalgia and has been shown to improve pain, sleep and quality of life but to be ineffective against depression [13]. As such, its clinical profile could be complementary of trazodone's clinical profile, which lacks analgesic activity but has an antidepressant effect. On the other hand, the mechanisms of action of both drugs are quite different, with trazodone exhibiting 5-HT_2_, α_1_, and H_1 _blocking properties and inhibiting serotonin presynaptic uptake, and pregabalin being a ligand on the α_2_δ subunit of type P/Q calcium channels. Thus, the combination of both drugs may be considered as a rational polytherapy both from a pharmacodynamic and a clinical point of view.

Accordingly with the above-mentioned considerations, we designed a two-phase study intended to assess the effectiveness and tolerability of flexibly dosed trazodone in the treatment of fibromyalgia and to evaluate whether the addition of pregabalin to those patients who were partially responsive to trazodone had additionally improved fibromyalgia symptomatology. The results of the first phase of the study showed that trazodone therapy markedly improved sleep quality and also had a positive and statistically significant effect on fibromyalgia severity, depression, anxiety and pain interference with daily activities but it did not improve pain intensity itself; the most frequent and severe side effect associated with trazodone in our sample was tachycardia, which was reported by 21% of the patients [14].

The main objective of the present study was to evaluate whether pregabalin augmentation additionally improved fibromyalgia symptomatology. A secondary objective was to assess the efficacy and tolerability of trazodone in the subset of patients who completed the first phase of the study and accepted to combine pregabalin with trazodone.

## Methods

This was a two-phase study, which each phase 12 weeks in duration. During the first 12 weeks patients were treated with trazodone, and during the following 12 weeks pregabalin was added to trazodone treatment.

The study included patients diagnosed of fibromyalgia according to the American College of Rheumatology criteria [15] and who were willing to discontinue their currently prescribed treatment. They were referred to our unit by their physicians (general practitioners, rheumatologists and physicians from pain-management clinics). Patients who had been previously treated with either trazodone or pregabalin but failed to improve or did not tolerate either of these drugs were excluded, as were pregnant or lactating women. Every patient gave informed consent to participate in the study, which was approved by the Ethics Committee of the University of Granada. The trial registration number was NCT-00791739.

Before beginning the study, the patients were required to withdraw from their current pharmacological medications prescribed for fibromyalgia. Nonpharmacological treatments, herbal remedies, drugs used on a p.r.n. basis, or drugs prescribed for associated pathologies were allowed; as rescue medication for pain paracetamol, up to 4 g daily, was recommended as the drug of choice but NSAIDs or tramadol were also permitted. After a patient-tailored washout period, trazodone was administered at a starting dose of 25 mg at bedtime and increased by 25 to 50 mg/day increments at two-week intervals if the patient reported little or no clinical improvement. The dosage increase was maintained until a relevant clinical benefit was reached or side effects appeared, the maximum allowed dosage being 400 mg daily. Pregabalin was administered at a starting dosage of 75 mg daily and dosage was increased, in 25 to 75 mg/day increments, at the same time intervals and under the same clinical criteria, with a maximum allowed dosage of 450 mg daily. During the second phase of the study, trazodone daily dosage remained unchanged, although if side effects potentially linked with the association of both drugs (i.e. sedation) were observed, it could be reduced. Patients were seen at baseline and at 2, 4, 6, 8, 10, 12, 14, 16, 18, 20, 22, and 24 weeks for dose adjustments. Clinical investigators were responsible for drug dispensation, dosage adjustments and adverse reactions monitoring.

Efficacy outcome measures included the Spanish-validated versions of the following scales: the Fibromyalgia Impact Questionnaire (FIQ) [16], the Pittsburgh Sleep Quality Index (PSQI) [17], the Beck Depression Inventory (BDI) [18], the Hospital Anxiety and Depression Scale (HADS) [19], the Brief Pain Inventory (BPI) [20], the Short-Form Health Survey (SF-36) [21], and a Patients' Global Improvement scale (PGI). Scales were administered at baseline and at weeks 6, 12, 18 and 24, with the exception of the SF-36, which was administered only at baseline and at weeks 12 and 24. Emergent side effects to trazodone and to trazodone plus pregabalin combination were recorded at each patient's visit by means of an open-ended question; patients were also instructed to phone the attending physician if they believed they were experiencing any drug-related side effect.

Because this phase of the study aimed to evaluate the usefulness of the trazodone plus pregabalin combination, the intention-to-treat (ITT) sample included those patients who had started pregabalin and had had at least a post-baseline assessment. Analysis of the ITT sample was performed with the last-observation-carried-forward approach (LOCF). Data were analyzed with repeated measures ANOVA. Effect sizes were calculated according to Cohen's formula and were considered small when lower than 0.50, moderate when ranging from 0.50 to 0.79, and large when equal to or greater than 0.80. Statistical analyses were performed using GraphPad Prism version 5.

## Results

**Figure 1:**
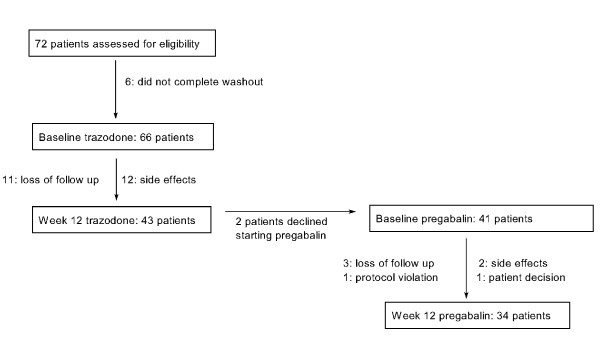
**Patients flow diagram**.

As it can be seen in Figure 1, 66 of the 72 screened patients started trazodone, and 43 of them completed the first 12 weeks of the study. Six patients were unable to complete the washout period complaining of worsening of their symptomatology. Of those, 41 patients who improved under trazodone accepted to receive pregabalin as add-on treatment. The 39 patients who had at least a post-baseline assessment after starting pregabalin constituted the ITT sample. Their demographic and clinical data are shown in Table 1. Twenty-nine patients withdrew from the study: 23 during the trazodone phase and 7 during the pregabalin phase; reasons for withdrawal are shown in Figure 1.

**Table 1:** Demographic and clinical data of patients at baseline

	(N = 41)
Sex (female/male)	39/2
Age (years)	(22-63)
(range and mean ± s.d.)	48 ± 10
Illness duration from diagnosis (years)	(1-19)
(range and mean ± s.d.)	46 ± 4.1
**Most frequent comorbidities [N (%)]**:	
Temporomandibular dysfunction	30 (73)
Migraine	19 (46.3)
Irritable bowel syndrome	13 (32)
Chronic fatigue syndrome	7 (17.9)
Thyroid disease	7 (17.9)
Rheumathoid arthritis	9 (23.1)
**Previous pharmacological treatments [N (%)]**:	
Paracetamol	22 (53.6)
NSAIDs	27 (65.8)
Tramadol	12 (29.3)
Benzodiazepines	25 (60.9)
Antidepressants	19 (46.3)
Anticonvulsants:	3 (7.3)
pregabalin/gabapentin	1 (2.4)
**Non-pharmacological therapies [N (%)]**	
Tai Chi	2 (4.9)
Swimming pool	2 (4.9)
Ozone therapy	1 (2.4)
Yoga	1 (2.4)
Physiotherapy	1 (2.4

Trazodone daily doses ranged from 50 to 300 mg with mean ± sd values of 199 ± 71 at week 12 and 194 ± 78 at week 24. Pregabalin daily doses ranged from 75 to 450 (324 ± 92) mg.

As shown in Table 2, FIQ total scores decreased steadily throughout the study period, with large effect sizes from weeks 12 to 24. Significant decreases were seen in most of the FIQ subscales scores, with large effects sizes in the work, pain, fatigue, morning tiredness and stiffness subscales at study endpoint. The improvement observed in the pain subscale of the FIQ under trazodone treatment was not seen in the mean severity scale of the BPI; however, a marked improvement in the mean interference of pain with daily activity of this test was apparent at both weeks 6 and 12 of trazodone treatment (Table 3).

**Table 2:** FIQ total scores and VAS subscales throughout the study (mean ± sd) (ES: effect sizes)

	baseline	week 6	week 12	week 18	week 24	P
Total scores	78.6 ± 11.5	72.2 ± 14.4	68.6 ± 16.6***	66.5 ± 18.1***	65.2 ± 18.5***	<0.0001
ES		0.56	**0.88**	**1.06**	**1.17**	
Work	8.19 ± 1.4	7.44 ± 1.9*	7.21 ± 2.1**	7.17 ± 2.1**	6.77 ± 2.4***	<0.0001
ES		0.54	0.71	0.73	**1.02**	
Pain	8.46 ± 1.4	7.92 ± 1.3	7.51 ± 1.8**	7.33 ± 1.09**	7.05 ± 2.3***	<0.0001
ES		0.38	0.70	**0.81**	**1.04**	
Fatigue	8.72 ± 1.2	8.02 ± 1.9	7.92 ± 1.6**	7.97 ± 1.7**	7.55 ± 2.3***	<0.0001
ES		0.58	0.67	0.62	**1.00**	
Morning tiredness	8.85 ± 1.2	7.64 ± 2.4	7.63 ± 2.4**	7.51 ± 2.3**	7.17 ± 2.4***	<0.0001
ES		**1.00**	**1.02**	**1.12**	**1.39**	
Stiffness	8.44 ± 1.7	7.55 ± 2.5**	7.26 ± 2.6**	7.15 ± 2.6**	7.06 ± 2.5***	= 0.0006
ES		0.52	0.72	**0.81**	**0.83**	
Anxiety	8.31 ± 1.7	7.47 ± 2.7	6.99 ± 2.5**	7.03 ± 2.4**	6.96 ± 2.4**	= 0.0006
ES		0.49	0.78	0.75	0.78	
Depression	7.90 ± 2.8	6.88 ± 2.9	7.01 ± 3.1	6.97 ± 2.7	6.85 ± 3.0	= 0.0754
ES		0.36	0.32	0.33	0.38	

*: p < 0.5, **: p < 0.01 and ***: p < 0.001 in relation to baseline (Tukey's post-test). Bold: large effect sizes.

**Table 3:** BPI, BDI and HADS scores throughout the study (mean ± sd) (ES: effect sizes)

	baseline	week 6	week 12	week 18	week 24	P
BPI mean severity	7.26 ± 1.6	6.69 ± 1.6	6.81 ± 1.8	6.33 ± 2-0	5.97 ± 2.3**	0.0028
ES		0.36	0.28	0.58	**0.81**	
BPI mean interference with daily activities	8.13 ± 1.6	6.90 ± 2.1**	6.60 ± 2.4***	6.48 ± 2.4***	5.93 ± 2.8***	<0.0001
ES		0.77	**0.95**	**1.03**	**1.38**	
BDI	28.0 ± 11.0	24.9 ± 13.1	21.5 ± 10.4***	19.1 ± 9.9***	19.3 ± 12.1***	<0.0001
ES		0.28	0.59	**0.81**	**0.79**	
BDI > 18 (N = 32)	31.2 ± 9.3	27.5 ± 12.7	23.2 ± 10.4***	20.4 ± 10.1***	20.8 ± 12.5***	<0.0001
ES		0.40	**0.87**	**1.16**	**1.12**	
HADS-A	13.9 ± 3.9	13.2 ± 4.5	12.0 ± 4.5*	11.9 ± 4.4**	11.5 ± 5.0**	= 0.0003
ES		0.17	0.48	0.52	0.61	
HADS-A > 7 (N = 37)	14.4 ± 3.4	13.7 ± 4	12.5 ± 4.1*	12.4 ± 4.0*	12.0 ± 4.6**	= 0.0008
ES		0.19	0.56	0.60	0.71	
HADS-D	12.2 ± 4.6	11.5 ± 4.7	10.6 ± 4.7	10.4 ± 5.0*	10.2 ± 4.9*	= 0.0061
ES		0.16	0.36	0.41	0.45	
HADS-D > 7 (N = 34)	13.3 ± 3.7	12.4 ± 4.2	11.2 ± 4.5*	11.0 ± 4.7**	10.7 ± 4.7**	= 0.0007
ES		0.25	0.58	0.63	0.71	

*: p < 0.05, **: p < 0.01 and ***: p < 0.001 in relation to baseline (Tukey's post-test). Bold: large effect sizes.

**Figure 2:**
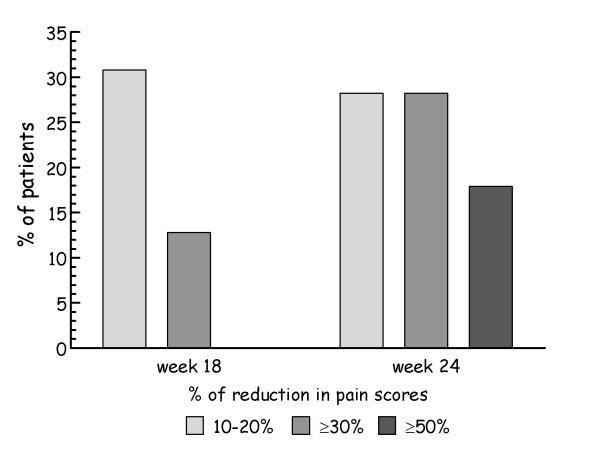
**Responder analysis of pain improvement in BPI severity scores**.

A responder analysis of the BPI mean severity scores was done according to the IMMPACT criteria, considering a decrease in pain scores of 10-20% as minimally important, a decrease ≥30% as moderately important, and a decrease ≥50% as substantial [22]. During the first phase of the study 12 (30.8%) of the 39 patients from the ITT sample had a minimally important decrease in pain scores, 5 (12.8%) patients had a moderately important decrease in pain scores, and no patient had a substantial decrease in pain scores. At the end of the study, after pregabalin addition, 11 (28.2%) patients had a minimally important decrease in pain scores, 11 (28.2%) patients had a moderately important decrease in pain scores, and 7 (17.9%) patients had a substantial decrease in pain scores (Figure 2).

PSQI total scores decreased markedly from baseline to week 12, and remained unchanged from week 12 to week 24 (Table 4). This pattern of reduction was apparent in most of the PSQI subscales, with particularly large effect sizes in sleep quality, sleep duration and sleep efficiency. The sleep disturbance was the only PSQI subscale that improved significantly after pregabalin addition, with relevant reductions between weeks 12 to 24 (p < 0.05).

**Table 4:** PSQI total and component scores throughout the study (mean ± sd) (ES: effect sizes)

	baseline	week 6	week 12	week 18	week 24	P
Total scores	16.0 ± 3.2	12.4 ± 4.0***	11.0 ± 4.6***	11.2 ± 4.4***	11.1 ± 5.1***	<0.0001
ES		**1.19**	**1.56**	**1.50**	**1.53**	
Sleep quality	2.54 ± 0.5	1.67 ± 0.8***	1.46 ± 0.8***	1.44 ± 0.8***	1.53 ± 0.9***	<0.0001
ES		**1.74**	**2.16**	**2.20**	**2.02**	
Sleep latency	2.51 ± 0.7	2.10 ± 0.9	1.80 ± 1.1***	2.08 ± 1.0*	1.87 ± 1.0***	<0.0001
ES		0.59	**0.99**	0.61	**0.89**	
Sleep duration	2.56 ± 0.6	1.90 ± 1.0***	1.56 ± 1.1***	1.51 ± 1.2***	1.54 ± 1.2***	<0.0001
ES		**1.10**	**1.66**	**1.75**	**1.72**	
Sleep efficiency	2.62 ± 0.7	1.92 ± 1.2***	1.72 ± 1.3***	1.69 ± 1.2***	1.62 ± 1.2***	<0.0001
ES		**1.00**	**1.27**	**1.33**	**1.41**	
Sleep disturbance	2.33 ± 0.5	1.97 ± 0.6	1.85 ± 0.6**	1.97 ± 0.7	1.44 ± 1.0***	<0.0001
ES		0.72	**0.90**	0.72	**1.68**	
Sleep medications	1.41 ± 1.4	0.82 ± 1.3	0.51 ± 1.1**	0.62 ± 1.2*	0.72 ± 1.2*	= 0.0028
ES		0.42	0.62	0.56	0.48	
Daytime dysfunction	2.28 ± 0.6	2.00 ± 0.9	1.95 ± 0.9	1.92 ± 0.9	1.92 ± 1.0	= 0.0454
ES		0.47	0.51	0.55	0.55	

*: p < 0.05, **: p < 0.01, and ***: p < 0.001 in relation to baseline (Tukey's post-test). Bold: large effect sizes.

Both depression and anxiety scores decreased significantly during the study period especially among patients with clinically relevant depression and/or anxiety at baseline (Table 3). Effect sizes were large in BDI scores and moderate in HADS anxiety and depression scores from weeks 12 to 24.

Despite the improvements observed in fibromyalgia severity, sleep quality, depression and anxiety, the only domain of the SF-36 that showed a striking amelioration was bodily pain, although a significant amelioration, albeit with small to moderate effect sizes, was also seen in physical function, role physical, vitality and mental health (Table 5).

**Table 5:** SF-36 component scores throughout the study (mean ± sd) (ES: effect sizes)

	baseline	week 12	week 24	P
PF (physical function)	27.2 ± 18.4	29.2 ± 16.5	36.0 ± 21.4**	= 0.0079
ES		-0.11	-0.48	
RP (role physical)	2.56 ± 12.6	8.33 ± 25.2	11.54 ± 28.0*	= 0.0657
ES		-0.46	-0.71	
BP (bodily pain)	10.9 ± 14.4	18.2 ± 17.9*	24.2 ± 20.6***	<0.0001
ES		-0.51	**-0.92**	
GH (global health)	26.3 ± 17.9	28.4 ± 18.5	29.6 ± 17.4	= 0.4321
ES		-0.12	-0.18	
VT (vitality)	13.6 ± 13.4	18.7 ± 19.4	19.9 ± 18.6*	= 0.0301
ES		-0.38	-0.47	
SF (social function)	35.6 ± 27.4	41.0 ± 28.5	42.2 ± 29.9	= 0.2150
ES		-0.20	-0.26	
RE (role emotional)	19.7 ± 32.2	24.8 ± 38.8	29.1 ± 39.9	= 0.3293
ES		-0.16	-0.29	
MH (mental health)	31.5 ± 21.5	38.1 ± 21.3	42.8 ± 24.6**	= 0.0023
ES		-0.31	-0.53	

*: p < 0.05, **: p < 0.01 and ***: p < 0.001 in relation to baseline (Dunnett's post-test). Bold: large effect sizes.

No relationship was found between trazodone dosage at week 12 and the degree of improvement either in the FIQ, PSQI or BDI scores.

**Figure 3:**
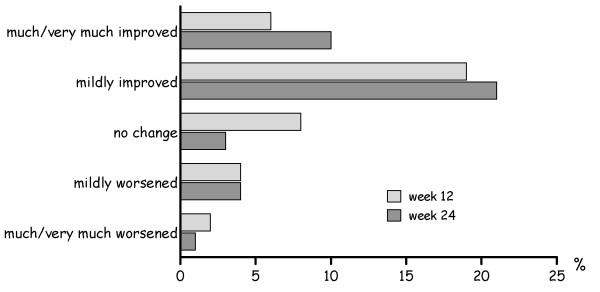
**Patients' Global Improvement scale at weeks 12 and 24**.

As shown in Figure 3, more patients reported to be better under the trazodone plus pregabalin combination than when receiving only trazodone.

**Figure 4:**
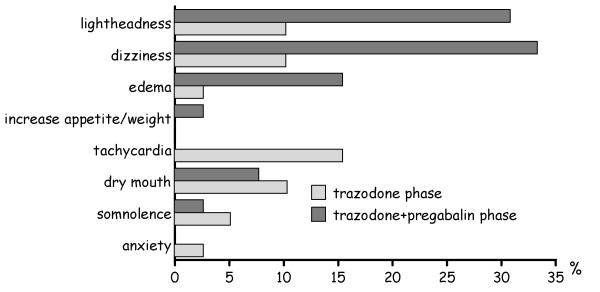
**Emergent adverse drug reactions during the study period**.

Thirty four (87%) patients reported treatment-related side effects during the study period, most of them being mild and transient. Tachycardia, dry mouth, dizziness and lightheadedness were those side effects more frequently reported during the trazodone phase of the study, whereas dizziness, lightheadedness, dry mouth and edema were the side effects more frequently reported with the trazodone plus pregabalin combination (Figure 4). Fourteen patients withdrew the study because of tolerability problems, 12 of them during the trazodone phase of the study and 2 of them during the trazodone plus pregabalin phase of the study; 10 cases of withdrawals were due to multiple side effects. There were no reported serious side effects.

## Discussion

Our data show that trazodone, in the range of doses administered during the study, not only improved sleep quality, but improved also fibromyalgia symptomatology, as shown by the decrease in the FIQ scores. Morning tiredness was the only FIQ subscale that was significantly improved with large effect sizes from the first evaluation of the study, probably reflecting the sleep-improving properties of trazodone. Depression and anxiety scores were also significantly improved at week 12, although large effect sizes were only seen in BDI scores. Trazodone treatment did not improve pain severity, as measured by the BPI, but it had a large impact on pain interference with daily activities, a fact that could also perhaps be attributed to the sleep-improvement properties of the drug. All these data paralleled those found when analyzing the data of all patients treated with trazodone, i.e., including those who did not enter in second phase of the study [14].

After the addition of pregabalin to trazodone, FIQ scores continued to decrease, with the most evident improvements appearing in morning tiredness, and the pain and anxiety subscales, reflecting the well-known sleep-improving, analgesic and anxiolytic effects of pregabalin. Sleep quality was not additionally improved beyond the amelioration observed during trazodone treatment, except for the sleep disturbance item the scores of which decreased significantly between weeks 12 and 24. The most relevant improvement seen after pregabalin addition was in bodily pain: the BPI mean pain severity score decreased significantly in relation to baseline, with a large effect size, and pain interference with daily activities additionally decreased beyond the initial improvement seen with trazodone.

It seems worthy of mention that the improvement in the bodily pain domain of the SF-36 was already significant at week 12, under trazodone treatment. This data possibly reflects the fact that, although trazodone did not have a significant impact on pain intensity, it allowed the patient to better tolerate bodily pain when performing customary activities.

Figure 2 shows how the percentage of patients reporting moderate and/or substantial decrease in bodily pain during trazodone treatment markedly increased during the second phase of the study, suggesting an enhancement in analgesia following pregabalin addition to the former drug. This observation is in agreement with the substantial improvement seen in the bodily pain component of the SF-36, and it is also consistent with pregabalin results in previous controlled trials [23,24].

The fact that 12 out of the total of 14 withdrawals due to adverse reactions occurred during the trazodone phase of the study could be indicative that the tolerability of trazodone is lower than that of pregabalin; in fact, the appearance of tachycardia was a major issue with trazodone, being reported by 14 (21%) patients and leading to withdrawal of 6 patients during the first phase of the study [14]. However, another explanation is also possible: it is well known that fibromyalgia patients are highly sensitive to drug side effects, and it seems likely that patients who withdrew due to tolerability problems during the first phase of the trial were those patients more prone to be intolerant of any drug.

Studies concerning drug combination in the treatment of fibromyalgia are scarce, and most of them are old. A double-blind, placebo-controlled study comparing amitriptyline, naproxen and a combination of both drugs found that either amitriptyline or amitriptyline plus naproxen were significantly better than placebo or than naproxen alone; however, the limited sample size of the study did not allow the authors to conclude that the minor differences between amitriptyline and amitriptyline plus naproxen were statistically significant [25]. A randomized, open-label study comparing low-dose cyclobenzaprine alone with cyclobenzaprine in combination with ibuprofen found that only morning stiffness improved significantly in the combination group in relation to the monotherapy group [26]. Another controlled study, on the contrary, found that cyclobenzaprine plus fluoxetine performed significantly better than cyclobenzaprine alone, despite the limited sample size evaluated [27]. Again, a double-blind crossover trial found that amitriptyline-fluoxetine combination improved pain scores and total FIQ scores more than either drug alone and more than placebo [28]. Our group added pregabalin to fibromyalgia patients already improved by quetiapine and found that pain and tiredness after awakening subscales of the FIQ, as well as the physical component of the SF-12, additionally improved with quetiapine plus pregabalin combination [29]; the results of this study were similar to those obtained with the combination of pregabalin and trazodone in the present study. Recently, an experimental study has shown, in an animal model of fibromyalgia, a potent antihyperalgesic effect of tramadol combined with milnacipran [30].

The above-mentioned data, with the exception of the two trials that evaluated a combination including an NSAID, and the results of which are consistent with the well-known fact that NSAIDs are not effective in the long-term management of fibromyalgia, suggest that the combination of two drugs with different mechanisms of action contributed to improving patients' outcomes. Hence, it is important to perform additional combination trials with drugs potentially synergistic and/or targeting different symptom domains.

Our results suggest that pregabalin treatment additionally improved the amelioration of fibromyalgia symptomatology induced by trazodone, especially in relation to physical pain. However, due to the uncontrolled design of our study, it is not possible to ascertain whether this improvement was due solely to the combination of both drugs or if the longer period of treatment also had a significant influence on the outcome.

As stated above, the main limitation of our study was the lack of a control group which was due to the fact that no financial support was found to perform a clinical trial. Trazodone is a relatively old drug without economic interest for the pharmaceutical industry, which, in addition, it is not usually interested in sponsoring combination drug trials. For this reason, the study was conceived as a preliminary research aiming to evaluate the potential usefulness of an old drug, widely used but scarcely investigated, in the management of fibromyalgia, as well as to assess whether its combination with pregabalin, which has been demonstrated to relieve pain in fibromyalgia patients, had an additive or synergistic effect on fibromyalgia severity. This design of the trial did not allow for controlling for a potential placebo effect; thus, the effectiveness of the treatment could have been overestimated, as it has been frequently observed when comparing non-controlled and controlled trials [31].

Another relevant limitation of our study was the restricted sample size. The recruitment period ranged from September 2006 to July 2007 and our objective was to include a greater number of subjects. However, many of the contacted patients were not willing to give up their previously prescribed medications and could not be included in the trial.

Thirty-two (48%) of the 66 patients who started trazodone treatment did not complete the evaluation period. Twenty-three of these patients withdrew during the first phase of the study, 12 (52%) due to treatment-emergent side effects; the remaining 11 withdrawals were losses of follow up that could also have been due to trazodone intolerance. Twelve (52%) of these 23 cases withdrew during the first four weeks of the therapy [14]. This high rate of early dropouts seems indicative of early intolerance to the drug, suggesting that treatment with trazodone should be closely monitored during the first weeks of administration.

A longer evaluation period with each drug would have allowed us to assess more thoroughly the effectiveness of both trazodone and of its combination with pregabalin. Nevertheless, this extension also would probably have facilitated the withdrawal of those patients who experienced only a marginal benefit with trazodone alone.

Our results could have been more striking if we had excluded patients with associated diseases. However, comorbidity in fibromyalgia is more the rule than the exception [4,8] and, in doing so, the data obtained would have been applicable to only to a very selective type of patients.

## Conclusion

Despite the limitations, we think that our data are indicative that the beneficial effects of trazodone on fibromyalgia clearly exceed its hypnotic activity and that the combination of trazodone with pregabalin can additionally improve patients' outcomes without increasing tolerability problems. Thus, we think that it will be worthy to evaluate, in controlled studies, whether the combination of pregabalin with trazodone, or another sleep-improving drug such as quetiapine or amitriptyline, is associated with a better long-term treatment outcome than either drug alone.

## Competing interests

- Dr. Fernando Rico-Villademoros is a freelance consultant who has received fees from Pfizer in the last year, although unrelated to the subject of the present study.

- The remaining authors declare that they have no competing interests.

## Authors' contributions

EPC, FRV and CMRL designed the study, evaluated the results, and wrote the manuscript. PMA and RML did the clinical work. All authors have read and approved the final manuscript.

## Pre-publication history

The pre-publication history for this paper can be accessed here:

http://www.biomedcentral.com/1471-2474/12/95/prepub
